# The quality of life of parents of children with down syndrome in a tertiary care hospital: A qualitative research study at Saudi Arabia

**DOI:** 10.1016/j.amsu.2022.104428

**Published:** 2022-09-05

**Authors:** Fatimah Saeed AlAhmari, Ahmed Fawzi Alageel, Maha Abdullah Aldosari, Muhammed Younus Bagha

**Affiliations:** a Developmental & Behavioural Pediatrics, King Abdullah Specialized Children Hospital, National Guard Health Affairs, Riyadh, Saudi Arabia; b College of Medicine, King Saud Bin Abdulaziz for Health Sciences, Riyadh, Saudi Arabia; c Mental Health Department, King Fahad Medical City, Riyadh, Saudi Arabia; d Department of Pediatrics, King Abdullah Specialized Children Hospital, National Guard Health Affairs, Riyadh, Saudi Arabia

**Keywords:** Down syndrome, Quality of life, Survey, Caregivers, Morbidity

## Abstract

**Background:**

As children with down syndrome (DS) usually have significant morbidities, they can also represent a significant burden on their caregivers and impact their quality of life (QoL). We conducted this study to investigate whether or not having DS children can impact the different domains of the QoL of their caregivers in Saudi Arabia.

**Methods:**

This is a cross-sectional phenomenological qualitative research study that was conducted in a tertiary care hospital, Riyadh, Saudi Arabia. To assess the study outcomes, we used the WHOQOL-BREF to assess the different domains of the QoL.

**Results:**

We have included 261 caregivers to DS children that responded to our questionnaire. The mean (SD) scores for the WHOQOL-BREF domains were 84 (±15), 88 (±15), 41 (±10), and 105 (±24), including the physical, psychological, social relations, and environmental domains, respectively. There was a significant difference between all of the scores that have been reported for these domains (P-value <0.001). Furthermore, educational level and the number of children were significantly associated with the psychological and physical domains, while the number of children was the only significant variable with the social relation. Finally, educational level, number of children, and average monthly income were all significantly correlated with the environmental domain.

**Conclusion:**

Our study indicates that the QoL of caregivers to DS children is significantly impacted in the different domains, indicating the urgent need to apply adequate interventions.

## Highlights

• Down syndrome is well-known for its less-than-optimal quality of life.
• The importance of caregivers and their roles in caring for down syndrome is important and is often overlooked.
• Studying and reporting the quality of life of the caregivers of Down syndrome patients is often missed and must be explored.

## 1 Introduction

Down syndrome (or trisomy 21) is known as a genetic disease that results as a disorder of an excess copy to trisomy 21. The prevalence of the condition is very high among the different global communities, and it has even been previously demonstrated to be the most common chromosomal-related disorder [1,2]. The disease severity of DS is hugely variant among the different patients. DS patients have a characteristic physical appearance, and manifestations generally include lifelong developmental delays and intellectual disabilities. Moreover, many complications can be associated with the disease, including hearing loss, heart defects, ear infections, eye diseases, and obstructive sleep apnea [[3], [4], [5]]. In addition to the significant morbidity that DS has on the affected children, it can also affect their caregivers's quality of life (QoL) [[6], [7], [8], [9]].

The term QoL is a broad one that is usually used to assess the different aspects of life. In another context, another term, which is health-related QoL (HRQoL), has also been used to specifically describe the different parameters of life, including psychological, physical, social, and emotional parameters that might be impacted by the patient's health [10]. Evidence in the literature indicates that caregivers to DS children have reduced mental health status, and usually require additional help to manage these children and enhance their psychological well-being [11,12]. Additionally, another investigation also indicated that caregivers to DS children have significantly higher stress levels as compared to others of normal children [13]. This is attributable to the fact that these children usually require high levels of care compared to other normal children, which can significantly increase the levels of stress and exhaustion among caregivers. Estimates indicate this by showing that emotional exhaustion was highly prevalent among caregivers to DS children [[14], [15], [16]]. Social embarrassment might also be an additional factor to the burdens that caregivers to DS children might face in their societies, which can also significantly impact their QoL.

QoL can also be impacted by the severity of their child's condition as some DS children might require excess care and medical services over others. Adequately assessing the domains of the HRQoL can help identify the most vulnerable groups with poorer health-related outcomes. Therefore, measuring HRQoL in caregivers to DS children might allow healthcare authorities to establish adequate interventional plans for this population to enhance the QoL-related outcomes. There is limited data about these outcomes in Saudi Arabia which makes it difficult for the local healthcare authorities to identify the populations in need. Therefore, we conducted this study to investigate whether or not having DS children can impact the different domains of the QoL of their caregivers in Saudi Arabia.

## 2 Methods

### 2.1 Study design and participants

This is a cross-sectional phenomenological qualitative research study that was conducted in a tertiary care hospital, Riyadh, Saudi Arabia. It included parents of children with DS using non-probability convenience sampling. The Rao soft website was used to calculate the sample size. Self-administered questionnaire on five points Likert scale was administered. We included parents of children that were diagnosed with DS that were 1) with or without medication 2) aged between 0 and 14 years old. We excluded caregivers if they were with 1) any child with DS that also suffered from another dual diagnosis, 2) other sick children in the family that needed care, and 3) caregivers that suffered from mental health conditions. The study was designed in accordance to the Standards for Reporting Qualitative Research [17]. It is registered with research registry with ID number: researchregistry8010.

The study design is a cross-sectional study of a self-administrative questionnaire, and parents were contacted at one time to have their answers to the questionnaire and the demographic factor sheet. There were a total of 1584 families with DS that were followed up in the tertiary care hospital from 2018 to 2019. We used a 95% confidence interval (CI), 5% margin of error, and therefore, the sample size was 310 families contacted.

### 2.2 Data collection

We used a self-administered questionnaire on five points Likert scale. Ethical approval was released from King Abdullah International medical research center (KAIMRC), and parents' consent was also obtained before taking part in the current investigation. Data were collected by measuring the parameters of the HRQoL in caregivers to DS children. The investigators were responsible for the process of collecting the data from the parents. The instrument used is the summarized version of a questionnaire developed by the world health organization (WHO) and was called the World Health Organization Quality of Life Scale (WHOQOL-BREF). The tool consists mainly of a total of 26, that were established to measure the following domains: psychological, environmental, physical health, and social relationships domains. It should be noted that the WHOQOL-BREF has been modified to be more suitable for application in clinical trials and other research investigations by being shorter than the original tool [18,19].

### 2.3 Statistical analysis

In the descriptive statistical analysis for different assessed domains, we used specific syntax, with transformed scores from zero to 100 [8]. Mean and standard deviation (SD) were used to represent continuous variables, while we used frequencies and percentages to represent categorical variables. The skewness and Kurtosis tests were used for testing the normal distribution of continuous variables. To compare different groups, we used the Friedman and Wilcoxon signed-rank tests. The Spearman rank correlation coefficient (rho) was used to determine the relationship between different variables [20,21]. Data were analyzed using R software version 4.1.1 using the packages (Rcmdr) and (corrr). The statistical significance was considered when the P-value was <0.05, for all tests.

## 3 Results

### 3.1 Baseline characteristics

We have finally included 261 caregivers to DS children that responded to our questionnaire. Among these participants, 124 (47.5%) were males, and 137 (52.5%) were females. Most of the included participants (62.8%) were >40 years of age. Regarding the educational level, 171 participants (65.5%) had high school and/or higher degrees, while only 22 (8.4%) participants were uneducated. Most of the included participants (64.4%) had more than four children (96.2%) were married, and (89.3%) were the parents to DS children. Besides, most participants (77.8%) had an average monthly income <14,000 SAR, and (67.8%) did not have medical problems. In Table 1, we have detailed these variables in addition to others.

**Table 1:** Socio-demographic profile of parents/caregivers of children and adolescents with Down syndrome (N = 261).

Variables		n	%
**Gender**	**Male**	124	47.5
	**Female**	137	52.5
**Age**	**Below 20**	3	1.1
	**20–29**	17	6.5
	**30–40**	77	29.5
	**Above 40**	164	62.8
**Educational Level**	**Uneducated**	22	8.4
	**Primary**	39	14.9
	**Elementary**	29	11.1
	**High school and above**	171	65.5
**Number of children**	**1–2**	36	13.8
	**3–4**	57	21.8
	**More than 4**	168	64.4
**Marital Status**	**Single**	4	1.5
	**Married**	251	96.2
	**Divorced**	5	1.9
	**Widowed**	1	0.4
**Caregiver (Person who cares for the child)**	**Parents (father or mother)**	233	89.3
	**Maid**	6	2.3
	**Grandparents**	2	0.8
	**Siblings**	9	3.4
	**Others**	11	4.2
**Type of Accommodation**	**Owner**	135	51.7
	**Tenant**	96	36.8
	**Rental agreement (installments)**	30	11.5
**Where do you live**	**District**	50	19.2
	**City**	211	80.8
**Average monthly income**	< **14,000 SAR**	203	77.8
	> = **14,000 SAR**	58	22.2
**Medical Problems**	**Yes**	84	32.2
	**No**	177	67.8

### 3.2 Correlation between WHOQOL-BREF domains

The mean (SD) scores for the WHOQOL-BREF domains were 84 (±15), 88 (±15), 41 (±10), and 105 (±24), including the physical, psychological, social relations, and environmental domains, respectively. There was a significant difference between all of the scores that have been reported for these domains (P-value <0.001) (Table 2). Besides, we have also assessed the association between the different domains of the WHOQOL-BREF. Based on these statistics, we found a significant association between social relationships and physical, psychological, and environmental domains (P-value <0.001), the physical and psychological domains (P-value = 0.021), the physical and environmental, and the and the psychological and environmental domains (P-value <0.001) (Table 3).

**Table 2:** Comparison among the results obtained for the WHOQOL-bref domains.

WHOQOL-bref	Physical	Psychological	Social relations	Environment
**Mean**	84	88	41	105
**Median**	84	92	40	108
**SD**	15	15	10	24
**Q1**	76	76	36	88
**Q3**	92	100	48	124
**n**	261	261	261	261
**P-value**	<0.001^a^			

WHOQOL = World Health Organization Quality of Life; SD = standard deviation; Q1 = 1st quartile; Q3 = 3rd quartile.

a Significant value – Friedman test.

**Table 3:** Pairwise comparisons between the WHOQOL-bref domains.

Sample 1-Sample 2	Test Statistic	Standard Error	Std. Test Statistic	P-value	Adjusted P-value^a^
**Social relations-Physical**	1.521	0.113	13.460	<0.001^b^	<0.001^b^
**Social relations-Psychological**	1.782	0.113	15.765	<0.001^b^	<0.001^b^
**Social relations-Environment**	−2.682	0.113	−23.732	<0.001^b^	<0.001^b^
**Physical-Psychological**	−0.261	0.113	−2.305	0.021^b^	0.127
**Physical-Environment**	−1.161	0.113	−10.273	<0.001^b^	<0.001^b^
**Psychological-Environment**	−0.900	0.113	−7.967	<0.001^b^	<0.001^b^

a Significance values have been adjusted by the Bonferroni correction for multiple tests.

b Significant values – Wilcoxon test.

### 3.3 Correlation between WHOQOL-BREF domains and population variables

Regarding the physical domain, a significant correlation was found between the estimated scores and educational level (P-value = 0.002) and the number of children (P-value = 0.025). Regarding the psychological domain, educational level (P-value = 0.002) and the number of children (P-value = 0.001) were also the only significantly correlated variables. Regarding the domain of the social relation, only the number of children was significantly correlated (P-value = 0.015), while other variables were not. Finally, educational level, number of children, and average monthly income were all significantly correlated with the environmental domain (P-value <0.001) (Table 4).

**Table 4:** Correlation between the WHOQOL-bref results and different variables.

Variables		Physical	Psychological	Social relations	Environment	Gender	Age	Educational Level	Number of children	Average monthly income
**Physical**	**Spearman's rho**	–								
	**P-value**	–								
**Psychological**	**Spearman's rho**	0.61	–							
	**P-value**	<0.001^a^	–							
**Social relations**	**Spearman's rho**	0.55	0.62	–						
	**P-value**	<0.001^a^	<0.001^a^	–						
**Environment**	**Spearman's rho**	0.59	0.67	0.57	–					
	**P-value**	<0.001^a^	<0.001^a^	<0.001^a^	–					
**Gender**	**Spearman's rho**	−0.16	−0.11	0	−0.05	–				
	**P-value**	0.01	0.083	0.956	0.417	–				
**Age**	**Spearman's rho**	−0.1	−0.04	−0.06	−0.08	−0.03	–			
	**P-value**	0.121	0.508	0.303	0.188	0.633	–			
**Educational Level**	**Spearman's rho**	0.19	0.19	0.08	0.27	−0.08	−0.37	–		
	**P-value**	0.002^a^	0.002^a^	0.180	<0.001^a^	0.191	<0.001^a^	–		
**Number of children**	**Spearman's rho**	−0.14	−0.2	−0.15	−0.21	−0.03	0.55	−0.38	–	
	**P-value**	0.025^a^	0.001^a^	0.015^a^	<0.001^a^	0.637	<0.001^a^	<0.001^a^	–	
**Average monthly income**	**Spearman's rho**	0.07	0.1	0.09	0.26	0.11	0.11	0.25	0	–
	**P-value**	0.276	0.109	0.138	<0.001^a^	0.079	0.076	<0.001^a^	0.946	–

WHOQOL = World Health Organization Quality of Life.

a Significant value P-value.

## 4 Discussion

In the present study, we aimed to assess the impact of having a child with DS on the QoL of their parents. Our results indicate that most of the included caregivers reported good satisfactory levels about their QoL. This is consistent with the findings of previous investigations from worldwide relevant investigations. Furthermore, many factors can contribute to the notable reduction in the QoL for parents to children with DS. These might include difficulty accepting their children's disabilities, altered family routines, and difficulty with getting the needed support [8,9]. However, it should be noted that although many issues and difficulties have been reported for caregivers to children with DS [22], the estimated QoL for the included population in the present investigation does not seem to be significantly impaired.

We have also assessed the levels of satisfaction per the different domains of QoL. Our results indicate a significant difference between the different domains, with the environmental domain having the highest total mean score, followed by the psychological, physical, and social relations domains. The reduced scores of these domains might contribute to the estimated reduction in the QoL among some of the included caregivers. This can be attributed to the potential burdens that having a child with DS induces on the psychological, social, and physical parameters of the caregivers [8,23,24].

In the same context, we also found that the number of children was the only significant variable to be associated with the social parameter of the QoL of the caregivers, indicating the high burden that having children with DS might represent for their caregivers, irrespective of other variables. The reported low social scores are usually secondary to the poor social performance of children with DS, which might be a direct cause for the embarrassment and anxiety of their caregivers [6,7]. Besides, it is logical that as a result of the reduced social performance of these children, caregivers are obliged to furtherly spend more time with their children and give them more attention. Moreover, evidence in the literature also shows that some caregivers reported that it is difficult to deal with the healthcare services for their children, which also takes over other responsibilities of these parents, leading to reduced QoL [7,25]. Accordingly, adopting social integration by establishing solid social networks with caregivers of children with DS can contribute to a beneficial resilience parameter to the affected caregivers [26,27]. However, it should be noted that not all caregivers can have access to such activities, which might not be affordable to many of them, and therefore, raising their children might also be a difficult practice and the reduced aspects of QoL. Accordingly, healthcare authorities should provide further facilitated access to these services, and provide home services to help these caregivers and enhance their QoL and social integration.

Although the environmental aspect of QoL of our population had the highest mean score compared to the other aspects, other investigations showed that it had the lowest scores among other domains [8,28]. Evidence shows that the environmental aspect of QoL is correlated with adequate access to healthcare services, leisure, and enhanced housing and transportation conditions [28,29]. Previous research also indicated the importance of leisure to caregivers of DS children to relieve the potential stress, and enhance the psychological and physical health. It can also reinforce the social integration between these caregivers and their families [30]. However, it should be noted that previous investigations reported that achieving this might be difficult in this population [31,32]. Educational level, number of children, and average monthly income were the only significant variables that were associated with the scores of the environmental domain of the QoL. The socioeconomic status and educational levels of the parents were also previously reported to be significantly correlated, and it has been demonstrated that it can significantly impact the association between DS children and their parents [8,[33], [34], [35]].

Our findings also indicate that psychological and physical aspects were significantly associated with the educational level and the number of children only. On the other hand, neither age, gender, nor monthly outcome was significantly associated. A previous investigation showed that caregivers with the highest monthly outcome had the highest QoL scores in all of the reported domains than others with lower monthly income [36]. On the other hand, another investigation by Gupta et al. [37] indicated that caregivers with higher socioeconomic levels had significantly increased care-related stress, which is probably attributable to a potential gap of the reality and expectations of caregiving among these parents. It is worth-mentioning that QoL of the carvings, in addition, be being significantly impacted by the cultural, economic, and social factors, it is also significantly affected by the spirituality and religious beliefs [38], which might explain the different findings among studies regarding the impact of the economic status on the psychological parameter because some caregivers might have adequate religious beliefs and educational levels that might enhance the levels of acceptance of their children. However, further investigations are still needed for further verification, and the current findings should be carefully interpreted.

Although our results indicate that age is not significantly associated with any of the QoL domains, previous studies indicated that it can significantly impact the different aspects of the QoL and can enhance the association between caregivers and their DS children [39,40]. This might be because older mothers might be mature enough to deal better with their DS children without adversely impacting their QoL domains. We also found that all of the QoL aspects were correlated with each other. A previous investigation aimed to assess the QoL of mothers to DS children and indicated that caregivers' psychological health could be significantly impacted by the maladaptive behaviors, on the condition that stress was present in the affected caregivers [15]. Other investigations also indicated that the physical health of mothers to DS children was associated with being optimistic about the development and health status of these children [8,39,41]. Accordingly, this can be considered significant evidence about the significant association between the different aspects of the QoL for caregivers to DS children as indicated by the results of these investigations and our reported findings.

It is worth mentioning that our study has some limitations. First, the sample size of the study is small, and the study has been conducted at a single center which might limit the generalization of the results. Second, the design of the study is cross-sectional, which might limit the ability to successfully follow the evaluation and adequately assess the association between the different aspects of the QoL of the included caregivers and the different variables, and therefore, a longitudinal investigation might have been more proper to investigate such outcomes. Finally, we also believe that comparing the current findings with others of a population of caregivers to non-DS children might have given a better insight into the significance of the current findings with this population.

## 5 Conclusion

Our study indicates that the QoL of caregivers to DS children is significantly impacted in the different domains. The average score for the environmental domain was the highest, while the average score for the social domain was the lowest. These findings indicate the importance of research that aims to identify the impact of DS on their caregivers and indicates the urgent need to offer adequate support to this population. Effective interventions should also be adequately planned by the healthcare authorities to enhance the development and health well-being outcomes of these children and their caregivers and elevate the QoL scores.

## Provenance and peer review

Not commissioned, externally peer-reviewed.

## Ethical approval

Institutional review board approval was obtained accordingly.

## Sources of funding

None.

## Author contribution

All authors contributed evenly to the conceptualization, drafting, data analysis, writing and proofreading of the research.

## Registration of research studies

-Name of the registry: Research Registry
-Unique Identifying number or registration ID: researchregistry8010
-Hyperlink to your specific registration (must be publicly accessible and will be checked):
-https://www.researchregistry.com/register-now#user-researchregistry/registerresearchdetails/62a9af4a159929001e45451c/

## Guarantor

Fatimah AlAhmari.

## Consent

Informed consent was obtained according and in guidelines of the declaration of Helsinki.

## Declaration of competing interest

The authors declare no conflict of interest.
